# Postprandial differences in the plasma metabolome of healthy Finnish subjects after intake of a sourdough fermented endosperm rye bread versus white wheat bread

**DOI:** 10.1186/1475-2891-10-116

**Published:** 2011-10-19

**Authors:** Isabel Bondia-Pons, Emilia Nordlund, Ismo Mattila, Kati Katina, Anna-Marja Aura, Marjukka Kolehmainen, Matej Orešič, Hannu Mykkänen, Kaisa Poutanen

**Affiliations:** 1 Department of Public Health and Clinical Nutrition. Clinical Nutrition, Food and Health Research Centre. University of Eastern Finland, Kuopio Campus. P.O. Box 1627, FIN-70211. Kuopio, Finland; 2 VTT Technical Research Centre of Finland. P.O.Box 1000, FI-02044. Tietotie 2, Espoo, Finland

**Keywords:** postprandial, rye bread, insulin, plasma metabolome, two-dimensional gas chromatography, gastric emptying rate

## Abstract

**Background:**

The mechanism behind the lowered postprandial insulin demand observed after rye bread intake compared to wheat bread is unknown. The aim of this study was to use the metabolomics approach to identify potential metabolites related to amino acid metabolism involved in this mechanism.

**Methods:**

A sourdough fermented endosperm rye bread (RB) and a standard white wheat bread (WB) as a reference were served in random order to 16 healthy subjects. Test bread portions contained 50 g available carbohydrate. *In vitro *hydrolysis of starch and protein were performed for both test breads. Blood samples for measuring glucose and insulin concentrations were drawn over 4 h and gastric emptying rate (GER) was measured. Changes in the plasma metabolome were investigated by applying a comprehensive two-dimensional gas chromatography coupled to time-of-flight mass spectrometry metabolomics platform (GC×GC-TOF-MS).

**Results:**

Plasma insulin response to RB was lower than to WB at 30 min (P = 0.004), 45 min (P = 0.002) and 60 min (P < 0.001) after bread intake, and plasma glucose response was significantly higher at time point 90 min after RB than WB intake (P = 0.045). The starch hydrolysis rate was higher for RB than WB, contrary to the *in vitro *protein digestibility. There were no differences in GER between breads. From 255 metabolites identified by the metabolomics platform, 26 showed significant postprandial relative changes after 30 minutes of bread intake (p and q values < 0.05). Among them, there were changes in essential amino acids (phenylalanine, methionine, tyrosine and glutamic acid), metabolites involved in the tricarboxylic acid cycle (alpha-ketoglutaric, pyruvic acid and citric acid) and several organic acids. Interestingly, the levels of two compounds involved in the tryptophan metabolism (picolinic acid, ribitol) significantly changed depending on the different bread intake.

**Conclusions:**

A single meal of a low fibre sourdough rye bread producing low postprandial insulin response brings in several changes in plasma amino acids and their metabolites and some of these might have properties beneficial for health.

## Background

Cereal foods are an important component of the daily diet throughout Western countries, and a major source of dietary carbohydrates. In a time of dramatically increasing prevalence of type 2 diabetes and associated diseases, tailoring of foods with slowly digestible carbohydrates and beneficial effects on glucose and insulin metabolism is of special interest for persons with impaired carbohydrate tolerance [1].

A repeatedly lowered postprandial insulinemia has been suggested to protect against obesity and to prevent excessive energy intake [2]. In our previous studies, rye bread, which has tradition of use as whole grain bread in northern Europe, has produced beneficial effects on postprandial insulin responses in healthy Finnish subjects [3] and in persons with metabolic syndrome [4] as compared to white wheat bread or whole-meal wheat and oat bread. These results have been recently confirmed by a Swedish research group [5,6]. However, the mechanism behind the lowered postprandial insulin demand after rye bread intake is not known.

Metabolomics is considered a promising tool for nutritional studies [7,8]. It aims to profile all low-molecular weight metabolites that are present in biological samples to enhance the understanding of the effect of a particular stimulus on metabolic pathways [9]. However, the application of metabolomics to biological samples obtained from cereal studies is still scarce. In animal studies, ^1^H-NMR-based metabolomics have revealed significant changes in some amino acids (AA) and AA-related metabolites after feeding rats [10] and pigs [11,12] with different whole grain diets. In humans, a recent study using an ultra-performance liquid chromatography coupled to time-of-flight mass spectrometry (UPLC-TOF-MS) platform has shown changes in the lipidomic profile of subjects with metabolic syndrome after a 12-week dietary intervention with rye bread versus wheat-oat bread [13].

The main aim of this study was to use the metabolomics approach to identify potential metabolites related to amino acid metabolism that might be involved in the postprandial insulin response after sourdough fermented endosperm rye bread intake.

## Methods

### Subjects

The subjects were recruited for the study through an intranet announcement among the personnel and students at the University of Eastern Finland in the autumn 2009. A total of 29 candidates were interviewed about their medical history, dietary habits, and physical activity. People with any food intolerances or allergies, and those who were smokers, had modified their diet or exercise patterns during the past year to lose weight, or were on medication (except oral contraceptives) were excluded. Sixteen subjects (13 female, 3 male) were eligible to participate in the study. There were no dropouts. All subjects had normal glucose tolerance at the time of entry to the study as determined by the oral glucose tolerance test (OGTT). The study protocol was approved by the Research Ethics Committee, Hospital District of Northern Savo. Each individual provided verbal and written informed consent before participation in the study.

### Test breads

The test breads were commercial refined endosperm sourdough rye bread (RB) (Leipomo Koskelonseutu Bakery, Suonenjoki, Finland) and commercial refined white wheat bread (WB) (Vehnäpaahto 500 g, Fazer Bakeries Ltd, Vantaa, Finland).

Content of protein by Kjeldahl method [14], total dietary fibre by enzymatic-gravimetric method [15], fat by the Flour-Mojonnier method [16], and digestible starch by the megazyme method [17] were determined from the test breads. Their moisture content was determined by oven drying at 130°C for 1 h. The major esterified and free phenolic acids (ferulic acid, *p*-coumaric acid and sinapic acid) were analysed using a method described by Mattila et al. [18]. *In vitro *hydrolysis of starch mimicking the mouth phase during bread digestion was performed as follows. Briefly, 100 mg of freeze-dried bread sample was pre-hydrated for 15 min at 40°C in 0.5 ml of 0.2 M acetate buffer, pH 5. Amylase (fungal source, Megazyme; dosage: 2.5 U/g freeze-dried bread sample) was added to the suspension and samples were collected at time points 0, 5, 10 and 15 min. Samples were centrifuged to remove the solids, after which content of solubilised starch in the supernatant was measured using the Megazyme method for digestible starch [17]. Subsequently, *in vitro *hydrolysis of protein of the breads was performed as described by Lappi et al. [19].

### Study design

The subjects fasted 10 to 12 h before each study visit. On the test morning, an intravenous catheter was inserted in the antecubital vein of the arm. After the fasting blood sample was taken the subjects received a test meal, which contained the test bread (50 g available carbohydrates), 0.30 g of 70% fat-margarine containing 100 μL of ^13^-C octanoic acid (a marker of the gastric emptying rate), 40 g cucumber, and 0.3 L of a non caloric drink (Fun Light, Felix Abba, Kumla, Sweden). Eight blood samples were taken after the start of eating the test meal (15, 30, 45, 60, 90, 120, 180 and 240 min).

The test bread portions were served in random order at intervals of 1-2 wk. Subjects were advised to maintain their habitual diet and exercise routines throughout the study. Heavy exercise, unusual large portions of food, and high intakes of fibre were prohibited on the day before the study visit, as well as alcohol consumption for 2 days before the test day.

Data on food consumption was collected using 24-h food records before each study visit, and nutrient intakes were calculated by using the MICRO-NUTRICA 2.5 Software (Finnish Social Insurance Institution, Turku, Finland).

### Biochemical analyses

Blood samples were taken using standard operational procedures within the Department of Public Health and Clinical Nutrition. Plasma insulin and plasma glucose were analyzed for each of the 9 time points. Plasma glucose was analyzed by using the glucose dehydrogenase enzymatic photometric assay (Konelab 20XTi Clinical Chemistry Analyzer, Thermo Electron Corp, Vantaa, Finland). Plasma insulin was analyzed by a chemiluminescent immunoassay (ADVIA Centaur Immunoassay System, Siemens Medical Solutions Diagnostics, Tarrytown, NY, USA).

Maximum increases in glucose and insulin concentrations were calculated by subtracting the highest value of each from the corresponding fasting value. Glucose and insulin areas under the curve (AUC) were calculated from the area beneath the curve above the fasting level including only the area before the concentration dropped below the fasting level by using GraphPad Prism 4.0 for WINDOWS (GraphPad Software, Inc., San Diego, CA).

### GC × GC-TOF/MS analyses

A total of 128 plasma samples, corresponding to the four time points 0, 15, 30, and 60 min, were analyzed by the GC × GC-TOF/MS method. Ten microliters of an internal standard-labeled palmitic acid (16:0-16, 16, 16 d_3_; 500 mg/L) and 400 μl methanol solvent were added to 25 μL of plasma. After vortexing for 2 min and incubating for 30 min at room temperature, the supernatant was separated by centrifugation at 10000 rpm for 5 min at room temperature. The sample was dried under constant flow of nitrogen gas. 25 μl MOX (2% methoxyamine HCl in pyridine) was added to the dried sample. The mixture was then incubated at 45°C for 1 h and derivatized with 25 μl of N-methyl-N-(trimethylsilyl)trifluoroacetamide (MSTFA) by exchanging acidic protons at 45°C for 1 h. 10 μl of retention index standard mixture with five alkanes at 800 ppm was added to the metabolite mixture. Sample order for analysis was established by randomization.

The instrument used was a Leco Pegasus 4D GC×GC-TOF mass spectrometer (Leco Inc., USA) with Agilent 6890N GC (Agilent Technologies, USA) and Combi PAL autosampler (CTC Analytics AG, Switzerland). The two-column GC×GC column set consisted of a 10 m × 0.18 mm × 0.20 μm RTX-5 (Restek Corp., USA) first dimension column, coupled to a 1.5 m × 0.10 mm × 0.10 μm BPX-50 (SGE, Australia) second dimension column (press fit connection). The GC oven was temperature programmed from 50°C (held for 1 min) to 295°C at 7°C/min (held for 3 min). The secondary oven operated 20°C above the primary oven temperature. The system operated at a constant pressure of 39.6 psi (helium as carrier gas). A modulation period of 5.0 s was used. All injections (1 μL) were performed in split mode (1:20) at a constant injector temperature of 260°C. The MS transfer line temperature was kept at 260°C, the ion-source temperature was held constant at 200°C, and the detector voltage was 1700 kV. Data were acquired at 100 spectra/s (45-700 amu).

Raw data were processed using ChromaTOF software (Leco) followed by alignment and normalization using an in house-developed software. Unwanted background peaks were eliminated using the classifications feature of of ChromaTOF software. In-house developed software was used to perform additional filtering using compound identification by ChromaTOF. In the end, the GC × GC-TOF/MS data set consisted of 255 metabolite peaks that passed quality control requirements [20].

Metabolites were annotated by using an in-house metabolite reference compound library that was created by analyzing standards for the compounds included in the library, as well as by searching in The Palisade Complete Mass Spectral Library, 600 K Edition (Palisade Mass Spectrometry). This library includes all spectra available from the NIST 2002 and Wiley registry collections as well as 150, 000 other spectra.

### Gastric emptying rate (GER)

Gastric emptying rate was measured using the ^13^C-octanoic acid breath test (^13^C-OBT).

Additional files show the detailed methodology used [see Additional file 1, ref. [21]] and a brief discussion of the obtained results [see Additional file 2, ref. [22-26]].

### Statistical analyses

Data were statistically analyzed using the SPSS statistical software (version 14.0, SPSS Inc, Chicago, IL) and R software version 2.4.1. Data are expressed as means ± SD. Data with skewed distribution were transformed logarithmically, but is presented in untransformed form. The normality of variables was tested by Kolmogorov-Smirnov test with Lilliefors correction.

For glucose and insulin AUCs, maximal responses, eating times, and diet analyses the statistical significance was assessed by the nonparametric Friedman's test followed by the Wilcoxon's test with Bonferroni correction for multiple comparisons (P < 0.05).

Relative changes for the metabolites detected by GC×GC-TOF-MS were calculated applying the so called rye and wheat indexes [27]. The indexes were calculated as follows:

Relative change x_ijk _= (Peak area x_ijk _at TP_j _- Peak area x_ijk _at baseline)/(median of peak areas x_ijk_) where x_ij _is the metabolite i (i = 1-255) measured for each subject j (j = 1-16) and bread k (k = 1 (rye bread), 2 (wheat bread). TP_j _refers to the time point at which the metabolite was measured (j = 15, 30, 60 min). Paired samples t-test for variables with normal distribution or Wilcoxon signed ranks test for abnormal variable distribution was used for comparisons within the groups. In order to correct for multiple comparisons, false discovery rate (FDR) q-value was calculated, with significance threshold set at recommended q < 0.10.

The sample size calculation for the present study was based on assuming a difference in the maximum increase in insulin response to wheat vs. rye bread of 10 ± 9 ml/l. With 80% chance of detecting the difference and using a two-sided significance level of 0.05, the minimum required number of subjects for the study was fifteen.

## Results

### Characteristics of the test breads

The RB portion contained more total fibre and phenolic acids, but less energy, less protein, and less fat than the WG portion at equal carbohydrate dose (Table 1).

**Table 1:** Nutrient composition of the test bread portions

	RB	WB
Portion size (g)	110.6	105.9
Energy (kcal)	230	291
Available carbohydrate (g)	50	50
Protein (g)	5.4	9.3
Fat (g)	0.9	4.5
Total dietary fibre	7.6	3.7
Moisture (g)	42.0	35.5
Phenolic acids^1^ ferulic acid sinapic acid *p*-coumaric acid	398 60 17	93 8 4

^1^μg/g of dry matter of bread: total amount, calculated from free and esterified amounts RB, refined endosperm sourdough rye bread; WB, refined white wheat bread.

**Figure 1:**
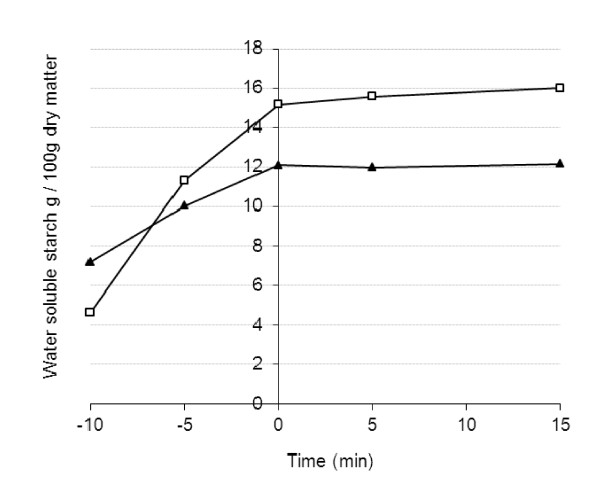
**Content (g/g dry matter of bread) of water extractable starch of the test breads after the in vitro amylase treatment mimicking the mouth phase (WB, Wheat bread (black triangle); RB, Endosperm rye bread(white circle))**.

**Figure 2:**
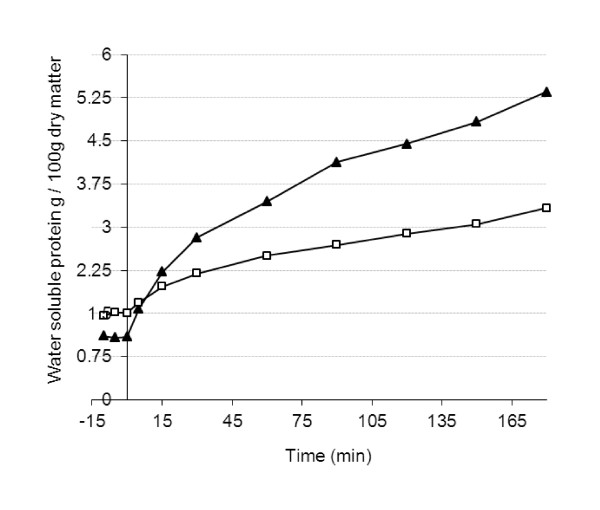
**Content (g/g dry matter of bread) of water extractable protein of the test breads after the in vitro pepsin treatment mimicking the stomach phase (WB, Wheat bread (black triangle); RB, Endosperm rye bread (white circle))**.

*In vitro *hydrolysis of starch and protein of the breads was performed using amylase and pepsin treatment to mimic hydrolysis reactions in both mouth and stomach. The starch hydrolysis rate was higher for RB than WB (Figure 1), but the hydrolysis of protein was slower for RB than WB (Figure 2). However, the relative amount of soluble protein at the beginning of the hydrolysis experiment (i.e. in untreated breads) was higher in RB than in WB. In the analysis of the hydrolysates (0-180 min) by SDS PAGE with the peptide separating gel (10-20% Tris-Tricine), RB had smaller molecular weight peptides than in WB both at the beginning of and during the *in vitro *hydrolysis (data not shown).

### Characteristics of the study subjects

The baseline characteristics of the subjects (13 women, 3 men) were the following: age = 23 ± 3.7 y, BMI = 22 ± 1.8 kg/m^2^, diastolic blood pressure = 115 ± 10.0 mmHg, systolic blood pressure = 73 ± 7.0 mmHg. The results for the OGTT were as follows: plasma glucose (0 min) = 5.1 ± 0.1 mmol/L and plasma glucose (120 min) = 5.0 ± 0.2 mmol/L. The weights of the subjects remained unchanged throughout the study, as did the mean energy, protein, fat, carbohydrate, starch and fibre intake calculated from the food records (data not shown).

### Postprandial plasma glucose and insulin responses

**Figure 3:**
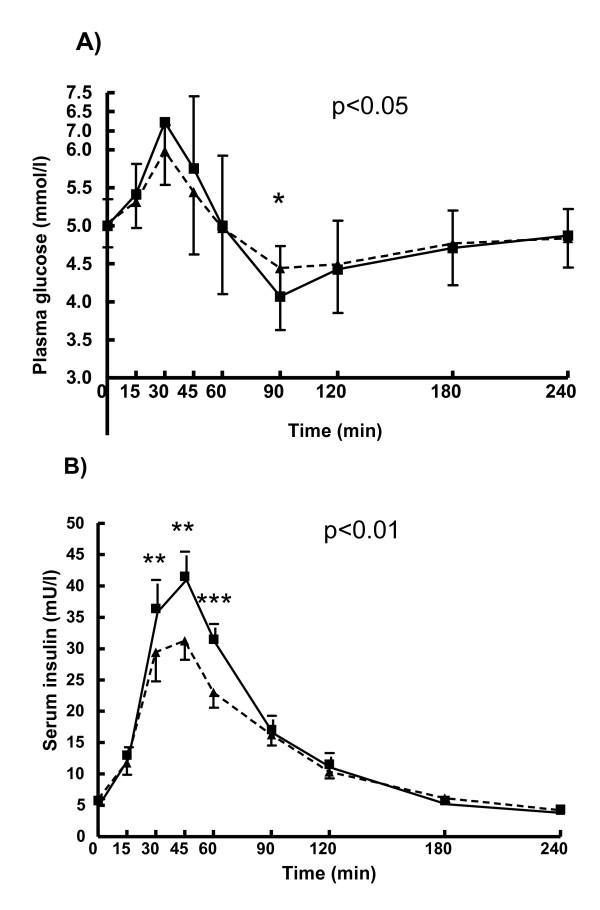
**Plasma glucose and serum insulin responses**. Nonparametric Friedman's test followed by the Wilcoxon's test with Bonferroni correction for multiple comparisons (P < 0.05). WB, Wheat bread (black triangle); RB, Endosperm rye bread (black square).

The maximum increase in glucose responses and the AUC_glucose _did not differ significantly among the breads (Figure 3). However, plasma glucose response was significantly higher at 90 min after eating of RB than WB (P = 0.045). Glucose concentration decreased below baseline fasting concentrations at 90 min after eating WB (P = 0.005).

Plasma insulin response to RB was lower than to WB at 30 min (P = 0.004), 45 min (P = 0.002) and 60 min (P < 0.001) after bread consumption (Figure 3). Furthermore, the maximum increase in insulin response to RB was significantly lower than the one for WB (28.8 ± 16.9 mmol/l vs. 38.9 ± 21.2 mmol/l; P = 0.001) and the AUC_insulin _significantly smaller (938 ± 515 mol/l × min vs. 1297 ± 511 mol/l × min; P = 0.04). The time intervals to reach the maximum increase in insulin response did not differ significantly after the intake of the breads.

### GC×GC-TOF-MS analyses

Two hundred and fifty-five metabolites were identified by the GC×GC-TOF-MS platform. Most of them were amino acids, organic acids, amides and aldehydes.

No significant changes were observed after 15 minutes of bread intake for any of the 255 detected metabolites. After 30 minutes of bread intake, which is the time point at which the difference in insulin response was significant for both breads, there were 26 metabolites showing significant postprandial relative changes (Table 2) (p and q values < 0.05). After 60 minutes of bread intake, the levels of three metabolites (phenylalanine, methionine and picolinic acid) remained significantly different from baseline after consuming the test breads (p and q values < 0.05) (Table 3).

**Table 2:** List of metabolites with significant postprandial relative changes after 30 min of test bread intake (rye versus wheat bread)

*Metabolite*	*Rye* *Index^1^*	*Wheat* *Index^1^*	*p* *value^2^*	*q* *value^3^*
**Metabolites that significantly decrease after RB and WB intake**				
Glutamic acid	-0.29	-0.59	0.047	0.004
Glucopyranose	-2.57	-0.03	0.039	0.026
Tyrosine	-4.97	-12.01	0.019	0.030
Octopamine	-7.06	-20.13	0.000	0.002
Monopalmitin	-19.65	-0.81	0.050	0.000
Myo-Inositol	-40.97	-36.65	0.014	0.010
Pyruvic acid	-47.36	-10.43	0.009	0.000
Gulonic acid-1, 4-lactone	-116.95	-90.07	0.000	0.000
**Metabolites that significantly increase after RB and WB intake**				
Benzeneacetic acid	1.54	1.36	0.000	0.002
Lysine	2.81	0.19	0.000	0.019
Alpha-ketoglutaric acid	4.41	15.15	0.028	0.008
2, 4-Dihydroxybutanoic acid	7.93	5.83	0.023	0.018
Propanedioic acid	94.10	38.17	0.050	0.015
2-Oxo-butanoic acid	323.09	182.23	0.023	0.008
**Metabolites that significantly increase after RB and significantly decrease after WB intake**				
Methionine	0.26	-0.18	0.019	0.001
Phenylalanine	0.57	-1.08	0.005	0.002
Threonic acid	1.29	-1.81	0.000	0.013
Ribitol	3.82	-2.37	0.030	0.042
Norvaline	5.22	-27.83	0.009	0.001
Citric acid	62.33	-77.12	0.029	0.003
**Metabolites that significantly decrease after RB and significantly increase after WB intake**				
Hydrocaffeic acid	-1.21	1.19	0.009	0.000
2-(Z)-Butenedioic acid	-1.44	0.01	0.050	0.024
Ascorbic acid	-1.94	7.44	0.028	0.033
Picolinic acid	-3.79	14.22	0.000	0.007
Norleucine	-7.05	2.76	0.021	0.001
Succinic acid	-8.10	5.79	0.040	0.021

^1 ^The rye and wheat indices were calculated as follows: Relative change x_ijk _= (Peak area x_ijk _at 30 min - Peak area x_ijk _at baseline)/(median of peak areas x_ijk_) where x_ij _is the metabolite i measured for each subject j (j = 1-16) and bread k (k = 1 (rye bread), 2 (wheat bread). ^2 ^Paired samples t-test. ^3 ^q-values were calculated from p-value distribution obtained from paired samples t-test.

**Table 3:** List of metabolites with significant postprandial relative changes after 60 min of test bread intake (rye versus wheat bread)

*Metabolite*	*Rye* *Index^1^*	*Wheat* *Index^1^*	*p* *value^2^*	*q* *value^3^*
**Metabolites that significantly increase after RB and significantly decrease after WB intake**				
Methionine	0.29	-0.16	0.010	0.003
Phenylalanine	0.86	-2.48	0.017	0.005
**Metabolite that significantly decrease after RB and significantly increase after WB intake**				
Picolinic acid	-3.82	10.07	0.018	0.013

^1 ^The rye and wheat indices were calculated as follows: Relative change x_ijk _= (Peak area x_ijk _at 30 min - Peak area x_ijk _at baseline)/(median of peak areas x_ijk_) where x_ij _is the metabolite i measured for each subject j (j = 1-16) and bread k (k = 1 (rye bread), 2 (wheat bread). ^2 ^Paired samples t-test. ^3 ^q-values were calculated from p-value distribution obtained from paired samples t-test.

Glutamic acid and tyrosine were the AAs that significantly decreased postprandially below their baseline values after 30 minutes for both RB and WB, while lysine increased after the same period. In the case of phenylalanine and methionine, their contents increased after RB, but decreased after WB intake, even 1 hour after consuming the test breads.

Interestingly, picolinic acid, a catabolite of the tryptophan metabolism, was found to be significantly decreased after RB and significantly increased after WB intake as well even 1 hour after consuming the test breads. On the contrary, ribitol, a precursor of tryptophan, significantly increased after RB and significantly decreased after WB after 30 minutes of bread intake.

There were also significant changes in some tricarboxylic acid cycle metabolites. Alpha-ketoglutaric acid levels significantly increased and pyruvic acid contents significantly decreased 30 minutes after intake of both test breads. While citric acid levels significantly increased after RB vs. WB intake, succinic acid showed the opposite trend.

Four organic acids (benzenacetic acid, 2, 4-dihydroxybutanoic acid, propanedioic acid, 2-oxo-butanoic acid) showed significant postprandial increases after 30 minutes of intake of both types of breads. The increases were always higher after RB than WB intake.

## Discussion

In the present study, we showed that RB consumption is followed by significantly different levels of various metabolites compared to those followed by WB consumption. Namely, levels of some amino acids, AA-derived compounds, TCA metabolites, and organic acids differed after intake of RB and WB. In addition we confirmed the earlier finding of lower postprandial insulin response to refined endosperm sourdough rye bread as compared to wheat bread [3].

The *in vitro *protein hydrolysis rate for the RB was slower than WB, and the relative content of soluble proteins before the hydrolysis was higher in RB than WB despite lower total protein content in RB. This finding, together with the different peptide profiles in the qualitative SDS-PAGE analyses of the test breads confirms that rye and wheat breads have different characteristics with regard to protein and peptide content. Consequently, we expected a different postprandial AA response. The analyses by GC×GC-TOF-MS confirmed significant postprandial changes in four essential AAs (phenylalanine, methionine, tyrosine and glutamic acid) during the insulin decrease. Neither one of them was branched-chain AA, which have previously shown to affect insulin signalling in adipose tissue [27].

However, significant changes in two metabolites related with the amino acid tryptophan were found in our study. On one side, ribitol, a metabolic end product formed by the reduction of ribose, significantly increased after RB intake while it decreased after WB intake. Interestingly, the level of this compound in fasting serum samples has been recently found to be significantly increased in post menopausal women after an 8-week consumption of high-fibre rye bread diet [28]. Since tryptophan is a precursor for the biosynthesis of serotonin which inhibits neuropeptide Y expression, thus depressing hunger [29], we hypothesize that increases in the tryptophan precursors ribitol and ribonic acid after the RB diet may mediate positive effects of higher tryptophan concentration, resulting in decreased appetite and food intake. This hypothesis would agree with recent postprandial studies that have shown that intake of rye is linked to a high subjective satiety [5,6,30]. On the other side, picolinic acid is a catabolite of the tryptophan metabolism reported as an activator of macrophage proinflammatory functions. This acid has shown to selectively induce the chemokines macrophage inflammatory protein-1alpha and -1beta in macrophages [31]. In our study the levels of picolinic acid significantly decreased after RB and increased after WB intake even one hour after consuming the test breads. This finding supports our earlier results linking rye bread consumption to inflammatory status. Higher inflammation markers were detected in fasting serum samples of subjects with metabolic syndrome after a 12-week diet with oat and wheat bread (high postprandial insulin response diet) than with rye bread and pasta (low postprandial insulin response diet) [4], in addition to higher lysophosphatidylcholine levels [13]. However, human studies are needed in order to confirm the inflammatory effects of picolinic acid reported in animal studies.

It is important to keep in mind that the physiological and biochemical response to a dietary perturbation is complex. The postprandial response *per se *depends on and involves multiple factors. Multiple processes related to metabolism, inflammation and oxidation are affected [32,33]. Recently, Pellis et al. [34] introduced a metabolomics and proteomics based postprandial challenge test to quantify the postprandial response of multiple metabolic processes in humans in a standardized manner. The application of the test in a dietary intervention study aimed at studying the effects of an anti-inflammatory supplement, revealed differences in several metabolites, some of them associated with amino acid and carbohydrate metabolism such as citric acid cycle metabolites. Differences in TCA cycle metabolites were also observed in our study after the intake of the two types of breads. However, these findings are probably not connected to the lower insulin response observed after RB intake. The larger decreases in pyruvic and succinic acids after RB meal might reflect a significant reduction in the aerobic glycolysis after RB intake. However, this should have contributed to a higher glucose response after RB than WB. Nevertheless, it is clear that the postprandial response might be a useful tool to reveal multiple aspects of metabolic health that would not be apparent from studying the homeostatic parameters at fasting state [34].

The rye bread tested in our study was baked by the traditional sourdough procedure which includes a fermentation process known to produce many changes in the bread matrix [35,36], and has a potential to reduce glycemic response [19,37]. In fact, lactic acid, either produced during sourdough fermentation or added directly to the bread, has been reported to reduce the rate of *in vitro *starch hydrolysis, suggesting that lactic acid interferes with the digestive process [38]. However, in our study the *in vitro *assays simulating the mouth phase digestion of starch of the test breads showed a higher initial starch hydrolysis rate for the RB than for WB. Nevertheless, since the *in vitro *starch hydrolysis was not monitored after the pepsin treatment in conditions mimicking the small intestine, precise conclusions about the effects of starch digestion cannot be made.

Bioprocessing has been shown to modulate the levels and bioaccessibility of bioactive compounds [39]. Recent studies demonstrated that sourdough fermentation increases the amount of free phenolic compounds [40], which have also been reported to have an impact on lowering the glycemic and insulin responses [41,42]. However, it is not likely that the higher phenolic acids content of the RB than in WB can explain the observed decreased insulin response after RB. Most phenolic acids in cereals are ester linked to polymers and require several biotransformations until they can exert their beneficial effects in the body. It is therefore very unlikely that they can have such acute postprandial effect on insulin metabolism as observed after the RB intake. On the other hand, lowered pH of sourdough bread compared with white bread is indicative of the increased level of organic acids in sourdough bread. It has been suggested that the presence of organic acids could reduce the GER [43]. However, the GER remained unchanged after both test breads although several organic acids identified by the metabolomics platform showed significant increases in their contents after RB versus WB intake.

There are, naturally, many other food factors already known or speculated to influence the postprandial glycaemia and insulinemia after bread consumption. These are for example differences in fibre content, botanical origin and structural properties of the raw material in cereal products that may have an effect on the postprandial responses. In addition, the type and extent of the bread processing and structure of the final product have been shown or speculated to have an effect on the postprandial responses [37,44]. In most of these studies, when insulin response is modified, usually glycemic responses are influenced in the same way, or vice versa. In the case of rye bread, however, the main difference from white wheat bread is the insulin response [3].

Our aim was to identify metabolites that are linked with the lowered insulin response after RB consumption in search for possible mechanism for this interesting response. We were unable to find molecules that might have an effect on lower insulin response, but we found other metabolites that could play a role in other 'rye-specific' effects shown by others such as higher satiety after rye bread consumption [6,28].

The finding that no significant changes in the metabolites were observed before 30 minutes, at which time point the insulin responses between RB and WB started to differ, does not support a causal explanation. Nevertheless, the changes observed in some metabolites associated with tryptophan were consistent with the changes in insulin levels after these breads and may suggest a beneficial inflammatory response after a single meal containing rye bread.

## Conclusions

Our study showed that a low fibre sourdough rye bread producing low postprandial insulin response brings in several changes in amino acids and their metabolites and some of these might have properties beneficial for health. However, we were unable to identify molecules that might have a direct effect on lower insulin response after rye versus white bread intake.

## List of abbreviations

AA: amino acid; AUC: area under the curve; BMI: body mass index; GC×GC-TOF-MS: two-dimensional gas chromatography coupled to time-of-flight mass spectrometry; GER: gastric emptying rate; OGTT: oral glucose tolerance test; RB: sourdough fermented endosperm rye bread; UPLC-TOF-MS: ultra-performance liquid chromatography coupled to time-of-flight mass spectrometry; WB: standard white wheat bread.

## Competing interests

The authors declare that they have no competing interests.

## Authors' contributions

KP, HM, MO, MK, IBP designed the research, IBP, EN, IM, KK conducted the research, IBP and EN analyzed the data, IBP, AMA, MK, HM, KP drafted and reviewed the manuscript. All authors read and approved the final manuscript.

## Supplementary Material

Additional file 1 **Determination of the Gastric emptying rate (GER) of the study participants**. The file contains the methodology used to determine the GER of the study participants by the ^13^C-octanoic acid breath test. Click here for file

Additional file 2 **Gastric emptying rate of the study participants after intake of rye and wheat bread**. The file contains the results obtained for the GER of the study participants after intake of rye and wheat bread. Results are briefly discussed. Click here for file
