# Supplementary material for: Postprandial differences in the plasma metabolome of healthy Finnish subjects after intake of a sourdough fermented endosperm rye bread versus white wheat bread
Source: Nutr J. 2011 Oct 19;10:116. doi: 10.1186/1475-2891-10-116 (PMC3214176; doi:10.1186/1475-2891-10-116)
Supplement: Additional file 1 — Determination of the Gastric emptying rate (GER) of the study participants. The file contains the methodology used to determine the GER of the study participants by the 13C-octanoic acid breath test. [file 1475-2891-10-116-S1.DOCX]

**Additional file 1**

**Determination of the Gastric emptying rate (GER) of the study participants**

Gastric emptying rate was measured using the ^13^C-octanoic acid breath test (^13^C-OBT).

Briefly, the ^13^C-labelled medium chain fatty acid, ^13^C-octanoic acid, is retained in the solid phase of the meal in the stomach. On entering the duodenum, rapid absorption, transportation to the liver and oxidation to ^13^CO_2_ occur. The rate at which ^13^CO_2_ appears in breath is considered as being determined by the GER. The GER of a subject is then rated to be rapid, normal, delayed or extremely delayed by calculating the kinetic parameters t_lag_ and t_1/2._

GER marker, 100 µL of ^13^-C octanoic acid, was mixed with 0.30 g of margarine and spread on one of the slices of the test bread. Four drops of vanilla flavouring (Spice& Food Finland Ky, Kuopio) were added to the slice in order to mask the characteristic flavour of the octanoic acid. The subjects were asked to eat first half of the slices without the GER marker, then the slice containing the GER marker, and then the rest of the slices without the marker.

Breath samples for ^13^CO_2_ measurement were collected in 1.5 L breath bags (Tecobag; Tesseraux Container, Bürstadt, Germany) every 15 min during the first 2 hours and thereafter in 30-min intervals. For each breath sampling, subjects were asked to take a deep breath in a stand up position, exhale briefly the air in the trachea, which is not thoroughly metabolised in the lungs, and briskly blow the rest of the air coming from inside the lungs into the sample bag. Otherwise during the 240 min- test period the subjects mostly stayed in a sitting position. Control samples were collected before the test meal.

The subjects attended a third visit in order to measure their habitual GER. The standard meal for this visit consisted of 100 µl ^13^C-octanoic acid mixed into a scrambled egg and served between two slices of wheat bread, and 150 ml of mineral water. Breath samples for ^13^CO_2_ measurement were collected as previously described.

Measurement of ^13^CO_2_ in exhaled air was performed by isotope-selective nondispersive infrared spectrometry (IRIS) with an infrared spectrometer (Wagner Analysen Technik, Worpswede, Germany). Breath samples were transferred directly from the breath bags into the measuring cuvettes by means of a pump. ^13^CO_2_ recovery was used to calculate values for GE kinetic parameters by using the two mathematical models described in the literature by Ghoos et al [21]. Calculations were performed with the IRIS-software v. 2.3. Two gastric emptying kinetic parameters were calculated: the time with maximum speed of gastric emptying after ingestion of the test meal (t_lag_ in min) and the half emptying time (t_1/2_ in min), described as the time when first half of the ^13^C-labelled substrate dose of the test meal has been metabolised.
