# Supplementary material for: Postprandial differences in the plasma metabolome of healthy Finnish subjects after intake of a sourdough fermented endosperm rye bread versus white wheat bread
Source: Nutr J. 2011 Oct 19;10:116. doi: 10.1186/1475-2891-10-116 (PMC3214176; doi:10.1186/1475-2891-10-116)
Supplement: Additional file 2 — Gastric emptying rate of the study participants after intake of rye and wheat bread. The file contains the results obtained for the GER of the study participants after intake of rye and wheat bread. Results are briefly discussed. [file 1475-2891-10-116-S2.DOCX]

**Additional file 2.**

**Gastric emptying rate of the study participants after intake of rye and wheat bread**

No significant differences were observed between the test breads with regard to the GER parameters (RB vs. WB: t lag (min) = 97 ± 20 vs. 104 ± 28; t ½ (min) = 151 ± 50 vs. 149 ± 39).

In addition to the main aim of our study, we tested the hypothesis that the different protein digestibility of the test breads would be reflected in a lower gastric emptying rate after intake of RB than WB. However, no significant differences were observed in the GER of the subjects by using the ^13^C-octanoic acid breath test. The ^13^C-OBT is a non-radioactive alternative to gamma scintigraphy, which is the most common method used to assess gastric emptying [22]. As far as we are concerned, this is the first study in which the ^13^C-OBT was applied to evaluate the effect of bread protein digestibility in GER. We previously reported no significant differences in GER after different intake of rye breads when paracetamol was baked into the test breads and its appearance in circulation was used as a marker for the GER [23]. The GER results of the present study are also in agreement with previous trials studying the effects of different breads on GER both by using the paracetamol method [24] and by ultrasound methods [25,26]. For instance, a recent assessment of GER by an ultrasound method revealed no differences in the GER after the ingestion of rye whole-meal bread compared to white wheat bread [26]. The paracetamol method has the inconvenience that the pharmacokinetics of paracetamol depends on the release and absorption of paracetamol across the small intestine varying within and between individuals. In contrast, with the ^13^C-OBT we controlled the within-subject variation by assessing the habitual GER of the subjects in a third visit. We are aware that the protein content in the test breads differed significantly in our study. However, we decided to fix the available carbohydrate content for both test breads instead of their protein content, as it allowed using similar bread portions in the postprandial test. In addition, the higher amount of fat in the WB than RB portion was expected to contribute in a significantly different GER after intake of both breads, because the fat content in foods is considered as a major contributor to the GER value. However, neither the different protein content nor the fat content of the test breads were able to contribute enough to show significant changes in the GER after the bread intake. Further studies with a higher number of volunteers should be carried out to confirm our results.
